# M&M: blending medicine and music

**DOI:** 10.20945/2359-3997000000161

**Published:** 2019-08-14

**Authors:** Marcello D. Bronstein

**Affiliations:** 1 Universidade de São Paulo Hospital das Clínicas Faculdade de Medicina Universidade de São Paulo São Paulo SP Brasil Editor-chefe da Archives of Endocrinology and Metabolism, chefe da Unidade de Neuroendocrinologia, Serviço de Endocrinologia e Metabologia, Hospital das Clínicas, Faculdade de Medicina da Universidade de São Paulo (HCFMUSP), São Paulo, SP, Brasil

It is always said that medicine is a combination of science and art: sometimes it’s more science, in other occasions it’s more art. This issue of AE&M provides a real demonstration on how these two conditions can be blended. Dr. Oscar Bruno, a distinguished Professor of Medicine in Buenos Aires (Argentina), worldwide acknowledged endocrinologist and an opera lover, wrote an outstanding essay about Amelita Galli-Curci, one of the most important coloratura sopranos from the first half of the 20^th^ century and that had her beautiful voice damaged after a thyroid surgery. (1) I am sure that our readers will be delighted by this article.
